# Supplementary material for: BRAPH: A graph theory software for the analysis of brain connectivity
Source: PLoS One. 2017 Aug 1;12(8):e0178798. doi: 10.1371/journal.pone.0178798 (PMC5538719; doi:10.1371/journal.pone.0178798)
Supplement: S1 Table — Means are followed by standard deviations. CTR, controls; PD-CN, Parkinson’s disease cognitively normal; PD-MCI, Parkinson’s disease with mild cognitive impairment. (DOCX) [file pone.0178798.s001.docx]

**S1 Table.** **Nodal degree for regions in Module III and V in PD-MCI subtypes and controls.**

| Module III regions | Controls (n=15) | Multiple-domain  PD-MCI (n=9) | Single-domain  PD-MCI (n=6) |
| --- | --- | --- | --- |
| Lh Superior Frontal G | 59.7 (0.8) | 58.0 (3.0) | 56.7 (4.7) |
| Lh Anterior Cingulate | 59.8 (0.6) | 58.0 (4.0) | 55.7 (5.9) |
| Lh Superior Frontal G | 58.7 (2.7) | 52.0 (13.9) | 53.6 (4.7) |
| Lh Middle Frontal G | 59.4 (1.5) | 55.7 (6.5) | 54.4 (7.5) |
| Lh Superior Parietal G | 59.7 (0.6) | 57.5 (4.8) | 56.4 (3.9) |
| Lh Inferior Frontal G | 59.1 (1.6) | 54.0 (7.0) | 56.3 (3.3) |
| Lh Precuneus | 60.0 (0.0) | 58.0 (4.9) | 56.6 (4.5) |
| Rh Superior Frontal G | 58.8 (1.8) | 53.2 (13.8) | 50.9 (8.7) |
| Rh Superior Parietal G | 59.0 (2.0) | 54.7 (5.8) | 53.4 (7.4) |
| Rh Precuneus | 59.7 (0.6) | 57.5 (4.8) | 56.4 (3.9) |
| Module V regions | Controls (n=15) | Multiple-domain  PD-MCI (n=9) | Single-domain  PD-MCI (n=6) |
| Lh Insula | 29.0 (0.0) | 28.8 (0.4) | 28.6 (1.3) |
| Lh Transverse Temporal G | 29.0 (0.0) | 28.7 (0.8) | 28.6 (1.3) |
| Rh Frontal Orbital G | 29.0 (0.0) | 28.5 (1.2) | 28.7 (0.7) |
| Rh Transverse Temporal G | 29.0 (0.0) | 29.0 (0.0) | 28.7 (1.0) |

Means are followed by standard deviations. CTR, controls; PD-CN, Parkinson’s disease cognitively normal; PD-MCI, Parkinson’s disease with mild cognitive impairment.
